# Separation and Detection of Microplastics in Human Exposure Pathways: Challenges, Analytical Techniques, and Emerging Solutions

**DOI:** 10.3390/jox15050154

**Published:** 2025-09-23

**Authors:** Asim Laeeq Khan, Asad A. Zaidi

**Affiliations:** 1 Department of Chemical Engineering, Faculty of Engineering, Islamic University of Madinah, Madinah 42351, Saudi Arabia; 2 Department of Mechanical Engineering, Faculty of Engineering, Islamic University of Madinah, Madinah 42351, Saudi Arabia

**Keywords:** microplastic separation, nanoplastic detection, biological matrices, vibrational spectroscopy, enzymatic digestion, microfluidic platforms, molecularly imprinted polymers, AI-aided spectral analysis

## Abstract

Microplastics (MPs) are increasingly recognized as widespread environmental contaminants, with confirmed presence in human tissues and biological fluids through ingestion, inhalation, and direct systemic exposure. Their potential impacts on human health have become an important subject of scientific investigation. The detection and quantification of MPs, particularly nanoplastics, in complex biological matrices remain challenging because of their low concentrations, diverse physicochemical properties, and interference from organic and inorganic matter. This review presents a critical assessment of current methods for the separation and detection of MPs from human-relevant samples. It examines pre-treatment, separation, and analytical approaches including physical filtration, density-based separation, chemical and enzymatic digestion, vibrational spectroscopy, thermal analysis, and electron microscopy, highlighting their principles, advantages, and limitations. Key challenges such as low sample throughput, absence of standardized procedures, and the difficulty of nanoplastic detection are identified as major barriers to accurate exposure assessment and risk evaluation. Recent advances, including functionalized adsorbents, improved anti-fouling membranes, integrated microfluidic systems, and artificial intelligence-assisted spectral analysis, are discussed for their potential to provide sensitive, scalable, and standardized analytical workflows. By integrating current challenges with recent innovations, this review aims to guide multidisciplinary research toward the development of reliable and reproducible detection strategies that can support MPs exposure assessment and inform evidence-based health policies.

## 1. Introduction

### 1.1. The Emerging Risk of Microplastic Contamination

Over the past century, the extensive use of plastic materials has supported significant industrial and technological progress; however, this development has also resulted in a long-lasting and large-scale environmental burden. Global annual production of plastics has surged, reaching 460 million metric tons in 2019, with projections suggesting this figure could approach 1.2 billion tons by 2060 [1]. This rapid increase in plastic production is compounded by an inefficient end-of-life waste management system, in which only about 9% of plastic waste is recycled, while the majority accumulates in landfills or is released into terrestrial and aquatic ecosystems [1]. Once in terrestrial and aquatic ecosystems, these materials are subjected to a range of degradation processes including photodegradation, mechanical abrasion, and biodegradation that fragment them into smaller particles [2]. This fragmentation gives rise to a class of contaminants known as microplastics (MPs), generally defined as plastic particles with a longest diameter of less than 5 mm [3]. Recent reviews have emphasized that microplastics in terrestrial ecosystems, originating from urban, agricultural, and manufacturing activities, are subject to complex transport pathways and show persistence across soil and landscape matrices [4].

These MPs are further categorized by their origin. Primary MPs are those manufactured at microscopic sizes for specific applications, such as microbeads in personal care products or industrial pellets [5]. Secondary MPs, which constitute the majority of environmental plastic debris, are formed from the breakdown of larger plastic items like bottles, bags, and textiles [6]. Owing to their small size and low density, these particles are readily transported across vast distances by wind and water currents, resulting in their widespread and persistent occurrence across diverse environmental matrices globally [7]. Microplastics have been reported in remote and extreme environments, from alpine mountain peaks to deep-sea sediments, although measured concentrations and particle types vary considerably depending on location and the analytical methods employed [1].

The extensive environmental distribution of MPs has made human exposure almost inevitable, a conclusion increasingly supported by biomonitoring studies. Emerging evidence from the scientific literature now confirms the presence of MPs particles within the human body. These synthetic contaminants have been detected in a diverse array of biological matrices, including blood, stool, semen, breast milk, sputum, and meconium, as well as in tissues associated with major organ systems such as the cardiovascular, gastrointestinal, respiratory, and reproductive systems [1]. This internal contamination, or “body burden,” is no longer a matter of speculation but a confirmed phenomenon, raising urgent questions about the potential consequences for human health.

Recent biomonitoring studies provide compelling evidence that MPs and nanoplastics (NPs) are not only present in the external environment but have already accumulated within the human body at physiologically relevant levels. For example, MPs have now been detected in human placenta, lung tissue, whole blood, breast milk, and semen, suggesting that exposure is both systemic and lifelong. Critically, emerging data from recent investigations confirm this body burden with increasing resolution, demonstrating polymer-specific distributions and particle size ranges down to the nanoscale [8]. Notably, a recent study by Bhat et al. [9] reported MPs in multiple human organ systems and highlighted their potential role in triggering oxidative stress and systemic inflammation, thereby reinforcing their relevance to public health risk assessment. These findings align with toxicological models showing that NPs (<1 µm) can cross epithelial and endothelial barriers, including the blood–brain and placental barriers, leading to unique toxicodynamic profiles not observed with larger particles [10,11]. Collectively, these new data underscore that the challenge is no longer to prove human exposure but to quantify it with sufficient precision to establish dose–response relationships. This shift emphasizes the urgent need for analytical strategies capable of resolving nanoscale particles, differentiating polymer chemistries, and linking particle burdens to mechanistic health outcomes.

While evidence from human epidemiological studies is still emerging, a growing body of toxicological investigations using cell cultures, organoids, and animal models indicates a wide range of potential adverse health impacts. Experimental exposure to MPs in these systems has been linked to oxidative stress, genotoxicity, chronic inflammation, metabolic disorders, immune dysfunction, neurotoxicity, and both reproductive and developmental toxicity [2]. However, the strength of evidence varies considerably across endpoints. Oxidative stress and pro-inflammatory signaling are consistently observed across in vitro and in vivo studies, suggesting they represent core initiating events in MPs-induced toxicity. In contrast, evidence for genotoxicity and neurotoxicity, while compelling, remains more fragmentary, often arising from high-dose exposures or specific polymer types, and requires further validation at environmentally relevant concentrations. Mechanistically, MPs and especially NPs can induce mitochondrial dysfunction and excessive reactive oxygen species (ROS) production, which in turn triggers DNA strand breaks and chromosomal aberrations, accounting for genotoxic outcomes. Similarly, the ability of nanoscale particles to cross the blood–brain barrier has been demonstrated in animal models, where subsequent neuroinflammation and altered neurotransmitter levels point to a plausible mechanistic basis for neurotoxicity.

This mechanistic appraisal highlights the need for more standardized studies that move beyond descriptive endpoints to interrogate causal pathways and dose–response relationships. The particles themselves can cause physical damage, while their large surface area allows them to adsorb and concentrate other environmental pollutants, such as heavy metals and persistent organic pollutants, acting as vectors for these toxicants into the body [12]. Together, these findings highlight the urgent need for reliable analytical methods to detect, identify, and measure the level of human exposure to these widespread synthetic particles

Despite the clear evidence of human exposure and the concerning toxicological data, the field’s ability to conduct a definitive human health risk assessment is severely constrained by a formidable analytical bottleneck [13]. A fundamental principle of toxicology and risk assessment is the establishment of a clear dose–response relationship, which necessitates precise quantification of the internal dose of a contaminant and its correlation with defined health outcomes [6]. For MPs, this is currently a substantial challenge. The fundamental problem lies in the immense difficulty of reliably separating and identifying these trace synthetic particles from the overwhelmingly complex biological matrices in which they are found [14]. Without robust and standardized analytical methods, the true internal dose in terms of particle number, mass, size distribution, and polymer composition remains largely unknown and incomparable across studies [6].

This methodological challenge initiates a persistent feedback loop that continues to hinder progress in exposure assessment and risk evaluation. The absence of reliable, high-throughput analytical tools prevents the large-scale, multi-center epidemiological studies needed to generate definitive human health data. In turn, the lack of conclusive human health data tempers the regulatory and financial impetus required to drive the development of the very analytical infrastructure that is needed. The scientific community is thus caught in a state of acknowledging a significant potential threat but lacking the tools to quantify its risk with the certainty required for decisive policy action. MPs are not a simple, single contaminant but a complex suite of materials varying in size, shape, polymer chemistry, surface properties, and adsorbed co-contaminants, a multidimensionality that makes their analysis extremely challenging [15]. Therefore, advancing the science of separation and detection is not merely a technical exercise; it is the critical, rate-limiting step toward understanding and mitigating the potential health risks of MPs pollution.

### 1.2. Scope and Structure of the Review

This review provides a critical and comprehensive evaluation of the current state-of-the-art in the separation and detection of MPs from biologically relevant human samples. Its primary objective is to synthesize existing knowledge, identify the most significant analytical challenges, and offer a forward-looking perspective on innovative solutions to overcome current limitations. The review begins by outlining the primary routes of human exposure and the types of MPs most relevant to health risk assessment, followed by the fundamental challenges of isolating these particles from complex biological matrices. A critical assessment of pre-treatment, separation, and analytical techniques is presented, highlighting their principles, strengths, and limitations. Key methodological gaps and trade-offs are also identified, including the lack of standardization and the persistent difficulty of detecting NPs. The review then explores promising directions such as smart adsorbents, advanced membranes, microfluidic systems, and the integration of artificial intelligence. By taking a systems-level view of the entire sample-to-answer process, this work aims to guide future research, foster multidisciplinary collaboration, and accelerate the development of robust, high-throughput analytical platforms essential for assessing and mitigating MPs-associated health risks.

**Figure 1:**
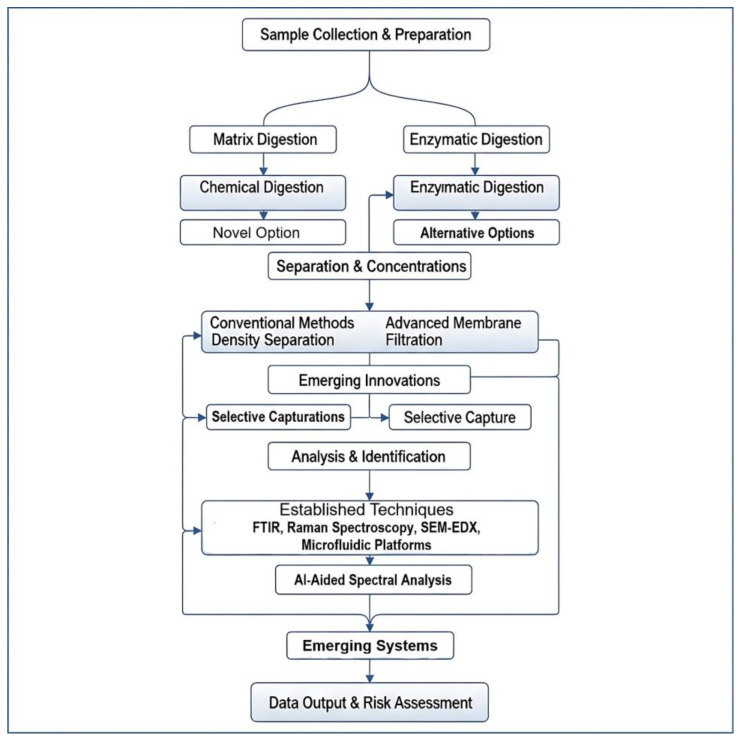
Conceptual framework of integrated workflows for MPs/NPs analysis in human biological matrices, from sample collection and digestion to separation, identification, AI-assisted analysis, and risk assessment.

This article was prepared as a narrative review, with a focus on recent advances in detecting MPs and NPs in human biological matrices. Bibliographic searches were conducted using PubMed, Web of Science, and Scopus, covering publications from the past two decades. The search strategy combined keywords such as MPs, NPs, human exposure, biomonitoring, toxicology, and analytical methods. Studies were included if they reported original data, reviews, or methodological developments relevant to the detection, quantification, or health impacts of MPs and NPs in human matrices. Exclusion criteria were applied to studies without clear relevance to human exposure, those limited to environmental monitoring without biomonitoring implications, or publications lacking peer review. Priority was given to peer-reviewed studies published in recent years to ensure the review reflects the most recent state of knowledge. Figure 1 represents the conceptual framework of integrated workflows for MPs/NPs analysis in human biological matrices.

## 2. Microplastic Exposure Pathways and Characteristics

Understanding the pathways by which MPs enter the human body is fundamental to assessing exposure and designing relevant analytical strategies. Humans are exposed to MPs through three primary routes: ingestion via the gastrointestinal (GI) tract, inhalation via the respiratory system, and dermal or direct systemic contact. Each pathway is associated with distinct sources, particle characteristics, and potential for translocation into the body.

### 2.1. The Gastrointestinal Tract: Ingestion as a Primary Route

Ingestion is widely considered the most significant pathway for human exposure to MPs [16]. MPs have been identified in a vast range of consumables, making oral intake a daily and unavoidable event. Drinking water, both from taps and particularly from plastic bottles, has been identified as a primary source, with some estimates suggesting that individuals may consume between 11,845 and 193,200 particles per year globally through this medium alone [3]. Seafood, especially filter feeding bivalves like mussels and oysters that are consumed whole, represents another major source, contributing an estimated 11,000 particles per person annually in European diets [16]. Other dietary staples, including sea salt, sugar, milk, and honey, have also been found to contain MPs [16].

Beyond direct contamination of food products, the packaging and containers used to store and serve them are significant contributors. Studies have shown that common polymer containers made of polypropylene (PP), polystyrene (PS), polyethylene (PE), and polyethylene terephthalate (PET) shed MPs particles into food, particularly when heated. For instance, individuals ordering take-out food 4–7 times per week may ingest up to 203 MPs pieces from the containers. Infant exposure is of particular concern; the sterilization and preparation of infant formula in PP bottles has been shown to release millions of MPs particles, leading to an estimated intake of 0.66 million particles for a bottle-fed baby in their first year. Furthermore, MPs contaminate agricultural systems through sewage sludge and plastic mulching, allowing them to be taken up by plants and enter the food chain through fruits and vegetables [16].

### 2.2. The Respiratory System: Inhalation of Airborne Particulates

Inhalation represents another major, and perhaps much ignored, route of exposure [2]. MPs are suspended in both indoor and outdoor air, originating from a variety of sources. A dominant contributor is the shedding of synthetic fibers from textiles, clothing, and carpets, with materials like polyester, nylon, and acrylic constantly releasing microscopic fibers into the environment [17,18]. Another significant source is the abrasion of larger plastic products, most notably tire and road wear particles, which are a complex mixture of synthetic rubber, polymers, and other additives [13]. Industrial activities, waste incineration, and landfill dust also contribute to the atmospheric load of MPs [17].

The fate of inhaled particles is largely determined by their aerodynamic diameter. Smaller particles and fibers can bypass the upper respiratory tract’s defenses and penetrate deep into the lungs, reaching the sensitive alveolar region [17]. It is from here that the smallest particles, particularly those in the NPs size range (<1 µm), are believed to have the potential to cross the alveolar–capillary barrier and enter the systemic circulation [17]. This pathway is supported by the detection of MPs in human lung tissue, sputum, and bronchoalveolar lavage fluid, confirming that the respiratory system is not only a site of exposure but also a portal for the systemic distribution of these contaminants [8]. Animal studies have further shown that inhaled PS particles can induce the expression of inflammatory proteins in lung tissue, highlighting the potential for localized toxicity [16].

### 2.3. Dermal and Other Systemic Exposure Routes

While ingestion and inhalation are the primary routes of environmental exposure, other pathways can contribute to the human body burden of MPs. Dermal contact with MPs is constant, arising from synthetic clothing, cosmetics, and dust. Although intact skin acts as an effective barrier to micrometer-sized particles, the possibility of particle penetration through damaged or compromised skin remains an active area of investigation [1]. The greatest concern for systemic exposure, however, comes from iatrogenic pathways, where medical procedures bypass biological barriers entirely.

A particularly alarming route of direct internal exposure is through medical treatments such as intravenous (IV) infusions. Studies have detected MPs ranging from 1 to 62 μm in filtered solutions from medical IV bags, estimating that thousands of particles could be delivered directly into a person’s bloodstream from a single 250-milliliter bag [19]. This represents a direct injection of foreign particulate matter into the circulatory system, a pathway with unknown but potentially significant health implications. Other medical devices and procedures may also serve as sources of contamination, highlighting a critical need to assess and mitigate MPs exposure within clinical settings [20]. The presence of MPs in such a controlled environment presents the truly pervasive nature of this contaminant.

**Figure 2:**
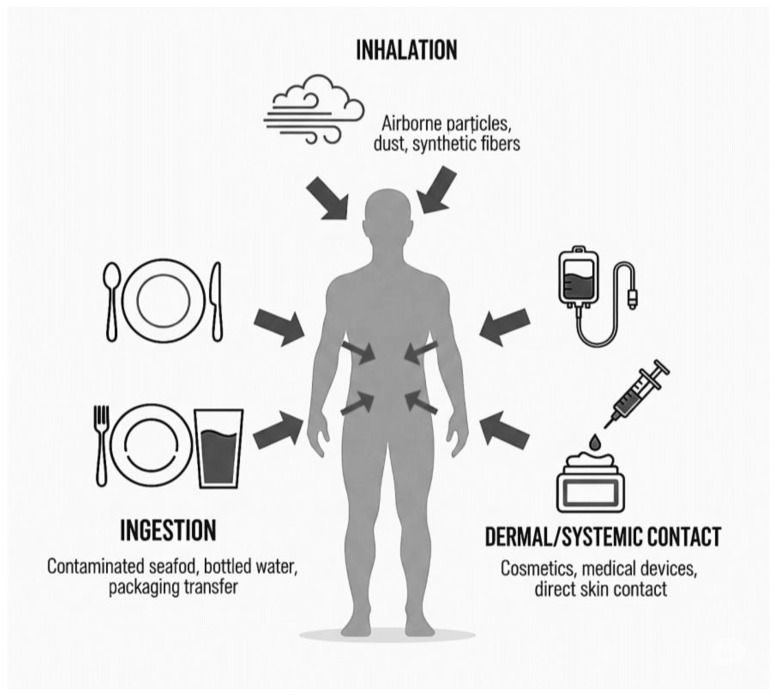
Primary routes of human exposure to plastic particles.

Figure 2 summarizes the three principal exposure pathways inhalation (airborne particles, synthetic fibers), ingestion (contaminated food, bottled water), and dermal/systemic contact (medical devices, cosmetics) by which MPs enter the human body. These pathways facilitate systemic distribution, contributing to internal accumulation.

### 2.4. A Profile of Human-Relevant Microplastics: Polymer Types, Morphologies, and Size Distributions

The MPs detected in human tissues are not a uniform contaminant but a heterogeneous collection of particles with diverse physicochemical properties. Analysis of human samples has revealed a wide range of polymer types, with the most commonly identified being those used in high-volume consumer goods and packaging. These include PE, PP, PS, PET and polyvinyl chloride (PVC), along with others like nylon (polyamide), alkyd resins, and polyurethane (PU) [12]. The morphology of MPs particles is highly diverse, encompassing irregular fragments, fibers, spheres, and films. Fibers, commonly derived from textile sources, and irregular fragments produced by the degradation of larger plastic items are the most frequently reported forms in human samples [21].

Particle size is arguably the most critical characteristic governing the biological fate and potential toxicity of MPs. The sizes of MPs found in human tissues span a vast range, from several millimeters down to the low-micrometer scale (e.g., 10 µm to over 4800 µm) [21]. However, it is widely recognized that smaller particles present a higher risk of crossing biological barriers and contributing to systemic exposure. Studies suggest that particles smaller than approximately 150 µm can be taken up by lymphatic tissue in the gut, while those smaller than 20 µm, and particularly those ≤10 µm, have the potential to cross biological membranes, enter the circulatory system, and distribute to distant organs and tissues [21]. This size-dependent ability to cross biological barriers is a critical determinant of potential toxicity, as it enables particles to migrate from primary entry sites such as the gastrointestinal tract or lungs to secondary organs, including the liver, kidneys, and even the brain, by traversing barriers like the blood–brain barrier [2].

To facilitate a comprehensive understanding of MPs exposure in humans, Table 1 provides an integrated overview of the predominant polymer types, particle sizes, morphological characteristics, and primary sources associated with each major exposure pathway. This synthesis, based on multiple data sources, serves as a valuable reference for researchers investigating the environmental and biological behavior of MPs.

## 3. Foundational Challenges in Microplastic Separation from Biological Samples

The accurate analysis of MPs in human tissues and fluids is predicated on the ability to first isolate them from their native biological environment. This separation step is not merely a procedural formality but represents the single greatest analytical challenge in the field. The inherent nature of biological samples presents a hostile and complex environment for the detection of trace synthetic polymers, creating a series of foundational obstacles that must be overcome to generate reliable data.

### 3.1. The Complexity of Biological MatriceS

Unlike relatively clean environmental matrices such as filtered water, biological samples are extraordinarily complex. Tissues from organs like the liver, lung, or placenta, fluids such as blood and breast milk, and excreta like stool are dense, heterogeneous mixtures of lipids, proteins, carbohydrates, nucleic acids, and minerals [5]. This complex biochemical environment presents a significant analytical challenge. MPs particles, as exogenous materials, are embedded within a matrix that is vastly more abundant and chemically heterogeneous, complicating their isolation and accurate characterization. Isolating these trace particles requires the near-complete removal of the surrounding biological components. This analytical challenge requires the selective removal of complex biological components while preserving the integrity and recovery of MPs particles present at trace levels [23]. The sheer complexity and variability of these matrices from the high lipid content of adipose tissue to the fibrous nature of lung tissue mean that no single separation protocol is universally effective.

The core of this challenge can be framed as a classic separation problem centered on mass balance. The goal is to design a process that maximizes the removal of the biological matrices (impurities) while simultaneously maximizing the recovery and preserving the integrity of the target analyte (MPs). Current methods often fail to strike this balance. Aggressive techniques that efficiently remove the matrix risk damaging the MPs, leading to an underestimation of their concentration and alteration of their physical characteristics [24]. Conversely, gentler methods may leave behind significant biological residues that interfere with subsequent analysis, leading to inaccurate identification or quantification [25]. The inability to effectively solve this mass balance problem for a wide range of polymers across diverse biological matrices is a primary source of the unreliability and poor comparability of data in the field.

### 3.2. Challenges of Trace-Level Detection Due to Low Microplastic Concentrations

The analytical difficulty is compounded by the fact that MPs are typically present at extremely low, or trace, concentrations within biological samples [23]. While the total particle count in an organ can be in the thousands, the cumulative mass of these particles is minimal relative to the total organ mass. This low concentration necessitates the processing of substantial initial sample volumes or masses to yield a sufficient quantity of particles for robust, statistically significant analysis [23]. For example, analyzing blood for MPs may require processing tens of milliliters, while tissue analysis may require several grams of material.

Handling large sample volumes further intensifies the difficulties posed by the complexity of biological matrices. A larger starting sample means a greater absolute amount of lipids, proteins, and other interfering substances that must be removed. This can easily overwhelm separation systems, leading to practical issues such as the clogging of filters and sieves, which not only slows down the workflow but can also lead to the physical loss of target particles [26]. This dynamic creates a difficult trade-off: a smaller sample may be easier to process but may not contain a representative number of particles, leading to a potential false negative, while a larger, more representative sample may be impossible to process effectively with current methods.

### 3.3. Interference from Biogenic Organic and Inorganic Matter

The biological matrix does not merely complicate separation; it actively interferes with the final analytical detection steps. Even after pre-treatment, residual organic matter can create significant analytical artifacts. Biogenic particles, such as cellulose fibers from diet or protein aggregates, can be easily misidentified as plastic particles during initial visual or microscopic inspection, leading to an overestimation of MPs counts and false positives [7]. This is a particularly acute problem for methods that rely solely on visual characterization, which is now widely considered unreliable without subsequent chemical confirmation [27].

Spectroscopic techniques, the gold standard for polymer identification, are also highly susceptible to matrix interference. During Raman spectroscopy, for instance, residual biological material can fluoresce when excited by the laser, producing a signal that is orders of magnitude stronger than the weak Raman scattering signal from the polymer. This fluorescence can completely overwhelm the target signal, making polymer identification impossible [27]. In Fourier Transform Infrared (FTIR) spectroscopy, strong absorption bands from residual organic matter (e.g., amide bands from proteins) can overlap with and obscure the characteristic absorption peaks of certain polymers. Therefore, the effective and nearly complete elimination of interfering biological material is essential to ensure accuracy and reliability in any analytical workflow [6].

### 3.4. Detection Challenges at the Nanoscale: Addressing the Analytical Limitations of Nanoplastics

The challenges of MPs analysis are magnified exponentially when considering NPs particles with dimensions less than 1 µm (or 1000 nm). These particles are of immense toxicological interest because their small size is predicted to facilitate translocation across cellular barriers, including the blood–brain and placental barriers [17]. However, the detection and characterization of NPs in complex biological matrices represent a largely unsolved analytical frontier [28]. At these size scales, conventional vibrational spectroscopy is fundamentally constrained by the diffraction limit, making individual particles below ~500–1000 nm extremely difficult to resolve. Even when advanced Raman or electron microscopy is employed, differentiating synthetic NPs from endogenous nanoparticles such as protein aggregates, lipoproteins, or extracellular vesicles remains a formidable challenge. These limitations frequently lead to underestimation of nanoparticle abundance, biased size distributions, and substantial quantification errors for particles <100 nm. As a result, the smallest and potentially most bioavailable fraction of the plastic burden is likely to be systematically overlooked, reinforcing the urgency of developing new analytical platforms capable of addressing this critical detection gap.

The fundamental limitations of current analytical instrumentation are a primary barrier. Most vibrational spectroscopy techniques, such as FTIR, are constrained by the optical diffraction limit, making them incapable of resolving individual particles much smaller than 10–20 µm [29]. While advanced Raman and electron microscopy techniques can visualize particles in the nano-range, their application to complex biological samples is exceptionally difficult [29]. The processes of separating and concentrating these particles from a biological digestate without significant loss are extraordinarily challenging. Moreover, the widespread occurrence of natural nanoparticles and macromolecular aggregates in biological systems significantly complicates the differentiation of synthetic NPs from background constituents. This analytical limitation, often referred to as the NPs detection gap, suggests that current studies may be overlooking a substantial and potentially the most toxicologically significant portion of the plastic particle burden within the human body [30,31].

**Figure 3:**
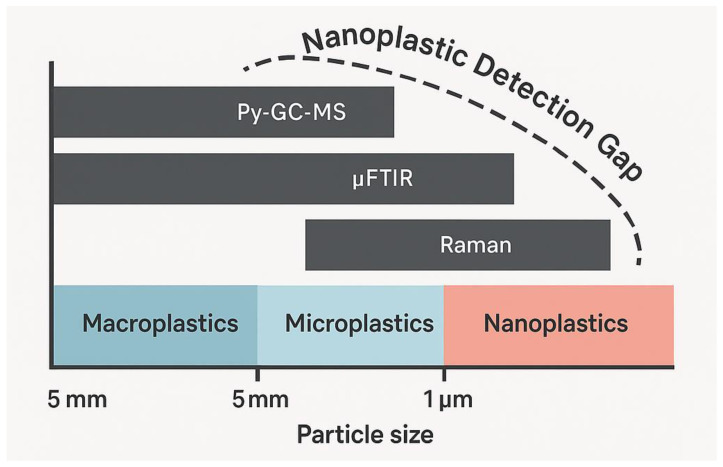
Detection capabilities of common analytical tools across particle size scales.

Figure 3 illustrates the significant gap in current analytical techniques for detecting nanoplastics, which remain largely beyond the detection limits of conventional methods such as Py-GC-MS and μFTIR. Although Raman spectroscopy extends further into the nanoscale, a substantial portion of the nanoparticle range remains analytically inaccessible, highlighting the urgent need for innovation in detection technologies.

## 4. Current Separation and Pre-Treatment Techniques

To address the challenges posed by complex biological matrices, researchers have developed a range of pre-treatment and separation techniques designed to isolate MPs for subsequent analysis. These methods can be broadly categorized into physical separation, density-based separation, and chemical or enzymatic digestion of the organic matrix. The choice of method involves a critical trade-off between digestion efficiency and the preservation of polymer integrity, and each approach carries its own distinct advantages and limitations.

### 4.1. Physical Separation: Filtration and Sieving

Physical separation methods, primarily sieving and filtration, are foundational steps in nearly every MPs analysis workflow [32]. Sieving, often performed with a series of stacked sieves of decreasing mesh size, serves as a preliminary step to remove large, non-target materials (e.g., large tissue fragments, hair) and to fractionate particles into different size classes [33]. This is particularly useful for processing samples like stool or digested tissues where a wide range of particle sizes is expected. However, sieving alone is insufficient for isolating MPs from the finely dispersed organic and inorganic components of a biological sample.

Filtration remains the principal method for concentrating MPs from liquid samples, generally applied following the digestion and solubilization of the major components of the biological matrix [34]. The liquid sample is passed through a filter membrane with a defined pore size, which captures the solid MPs particles on its surface for subsequent microscopic or spectroscopic analysis. A variety of filter materials are used, including glass fiber, polycarbonate, cellulose nitrate, and alumina, each with different chemical compatibilities and surface properties [34]. The selection of an appropriate pore size is critical and must be aligned with the data quality objectives of the study; smaller pores capture smaller particles but are more susceptible to clogging, a significant issue when dealing with residual organic matter from incompletely digested biological samples [26].

### 4.2. Density-Based Separation: Principles and Limitations

Density separation is a widely used technique in environmental science for separating MPs from denser inorganic matrices like sand and sediment [33]. The principle relies on immersing the sample in a high-density saline solution, such as aqueous solutions of sodium chloride (NaCl, density ~1.2 g cm^−3^), zinc chloride (ZnCl_2_, ~1.7 g cm^−3^), or sodium iodide (NaI, ~1.8 g cm^−3^). Most common polymers, such as PE and PP, have densities less than water and will float, while denser polymers like PS and PVC will float in the more concentrated salt solutions, allowing them to be separated from sinking mineral particles (e.g., quartz, density ~2.65 g cm^−3^) [33].

Despite its utility for environmental samples, density separation has severe limitations when applied to biological matrices. The primary issue is that many biogenic organic components, such as lipids and protein aggregates, have densities similar to or less than those of the plastic particles, meaning they will float alongside the MPs and contaminate the separated fraction. This makes the technique ineffective as a primary clean-up step for tissues, blood, or stool. Furthermore, the method is inherently incapable of recovering high-density polymers like PET (density ~1.38 g cm^−3^) and PVC (density ~1.4 g cm^−3^) when using common solutions like NaCl, as these plastics will sink with the unwanted matrix components, leading to a systematic under-reporting of their presence [33].

### 4.3. Chemical and Oxidative Digestion for Matrix Removal

To overcome the interference from organic matter, aggressive digestion methods are frequently employed to break down the biological matrix into soluble components. These methods can be highly effective, often achieving digestion efficiencies greater than 90% [35]. Common approaches include alkaline digestion with potassium hydroxide (KOH), which effectively saponifies lipids and hydrolyzes proteins, and is often used for digesting whole organisms or tissues like the placenta [24]. Acid digestion, using strong acids like nitric acid (HNO_3_), is also effective at destroying organic matter but is extremely corrosive. Oxidative methods utilize powerful oxidizing agents like concentrated hydrogen peroxide (H_2_O_2_) or Fenton’s reagent (a solution of H_2_O_2_ and an iron catalyst) to degrade biogenic material through radical oxidation reactions [24].

The primary and most significant drawback of these aggressive chemical treatments is their potential to damage the target MPs. The harsh chemical environments and often elevated temperatures used to accelerate digestion can alter or completely destroy certain types of polymers [27]. For example, alkaline solutions like KOH are known to degrade PET and PC, while strong acids can affect PA. Even oxidative treatments can cause discoloration or mass loss in polymers like PA, especially at higher temperatures [24]. This creates a critical analytical trade-off: the very process used to reveal the MPs may simultaneously be destroying a fraction of them, leading to biased and inaccurate quantification of the true polymer composition in a sample.

### 4.4. Enzymatic Digestion: As a Selective Approach to Preserving Polymer Integrity

In response to the destructive nature of harsh chemical treatments, enzymatic digestion has emerged as a gentler alternative for matrix removal. This approach utilizes specific enzymes to selectively catalyze the breakdown of biological macromolecules while leaving synthetic polymers unharmed. A mixture of enzymes can be tailored to the specific composition of the sample matrix. For example, proteases like proteinase K and trypsin are used to digest proteins in tissues and blood; lipases are used to break down fats; amylases target carbohydrates; and cellulase or chitinase can be used for plant material or invertebrate exoskeletons, respectively [25].

The advantage of enzymatic digestion is its high specificity, which results in excellent polymer recovery rates and preservation of the particles’ physical and chemical integrity [35]. Studies have shown that enzymes like trypsin can achieve good digestion efficiency (e.g., 88% for mollusk tissue) with no observable impact on a wide range of common polymers [36]. However, this approach is not without its trade-offs. Enzymatic methods are generally much slower, often requiring incubation periods of hours to days, and are significantly more expensive than simple chemical reagents [25]. Furthermore, their digestion efficiency can be lower than that of aggressive chemical methods, and for particularly complex or resilient matrices, a combination of enzymatic treatment followed by a milder oxidative step may be necessary to achieve complete sample clean-up [35].

### 4.5. Quality Assurance and Quality Control (QA/QC) in Pre-Treatment

Regardless of the chosen separation and digestion method, a rigorous Quality Assurance and Quality Control (QA/QC) program is essential to ensure the accuracy and reliability of the final data [7]. Due to the widespread presence of MPs especially fibers from textiles and fragments from laboratory materials, the potential for background contamination during sample processing is considerably high [7]. Without stringent controls, it is impossible to distinguish between MPs genuinely present in the original sample and those introduced during handling and analysis.

Key QA/QC measures include processing samples in a clean-air environment, such as a laminar flow hood, to minimize airborne contamination. All lab personnel should wear non-synthetic clothing, such as cotton lab coats, to prevent shedding of textile fibers. All glassware and equipment must be cleaned, rinsed with filtered ultrapure water, and covered with aluminum foil when not in use [37,38]. Most importantly, the inclusion of procedural blanks is non-negotiable [39]. These are samples containing only the reagents used in the procedure that are processed alongside the real samples. Any particles found in the blanks represent contamination and must be quantified and subtracted from the sample counts, or the entire batch must be discarded if contamination levels are too high. These measures are critical for generating defensible data in the field of trace contaminant analysis.

To support the selection of appropriate matrix removal strategies, Table 2 presents a comparative evaluation of commonly employed digestion methods for biological samples. Each method is assessed based on its underlying chemical mechanism, digestion efficiency, compatibility with polymer integrity, and practical considerations, including advantages and limitations. This synthesis serves as a valuable guide for researchers aiming to balance effective tissue degradation with minimal impact on MPs recovery.

## 5. Analytical Techniques Post-Separation

Once MPs have been isolated from the bulk of the biological matrix and concentrated, typically on a filter, the next critical step is their identification and quantification. This is accomplished using a range of sophisticated analytical techniques, each providing different types of information. Spectroscopic methods are used to determine the chemical identity of the polymer, thermal methods can quantify its mass, and microscopic techniques reveal its physical characteristics. No single technique is sufficient for comprehensive characterization; thus, a multi-method approach is often required.

### 5.1. Vibrational Spectroscopy: FTIR and Raman Microspectroscopy

Vibrational spectroscopy serves as a fundamental method for the identification of MPs, providing unequivocal confirmation of polymer type and composition. The two primary techniques are Fourier Transform Infrared (FTIR) spectroscopy and Raman spectroscopy [42]. Both methods probe the vibrational modes of chemical bonds within a polymer molecule. FTIR measures the absorption of infrared light at specific frequencies corresponding to these vibrations, generating a characteristic absorption spectrum that serves as a molecular “fingerprint” [29]. Raman spectroscopy achieves a similar outcome by measuring the inelastic scattering of monochromatic laser light, where shifts in the scattered light’s frequency correspond to the molecule’s vibrational modes [29]. When coupled with a microscope (µ-FTIR and µ-Raman), these techniques can analyze individual microscopic particles.

While both techniques provide complementary information, they have distinct strengths and weaknesses. FTIR is generally more robust, faster for large area mapping (especially with Focal Plane Array detectors), and benefits from extensive, well-established spectral libraries for polymer matching. However, its spatial resolution is limited by the diffraction of infrared light to approximately 10–20 µm, making it unsuitable for analyzing smaller MPs or NPs. Raman spectroscopy, using visible light lasers, offers a much higher spatial resolution, capable of analyzing particles down to ~1 µm and even smaller with advanced configurations, making it the preferred method for smaller particles [29]. Its major drawback is a susceptibility to fluorescence interference from residual organic matter or dyes within the plastic, which can obscure the weak Raman signal [27].

### 5.2. Thermal Analysis: Pyrolysis–Gas Chromatography–Mass Spectrometry (Py-GC-MS)

Pyrolysis–Gas Chromatography–Mass Spectrometry (Py-GC-MS) is a highly sensitive analytical technique capable of both identifying and quantifying polymeric materials, though it is inherently destructive to sample morphology. In this method, the sample containing MPs is rapidly heated to a very high temperature (e.g., 600–1000 °C) in an inert atmosphere [29]. This process, known as pyrolysis, breaks the polymer chains down into smaller, characteristic volatile fragments. These fragments are then swept into a gas chromatograph (GC) for separation, and subsequently into a mass spectrometer (MS) for identification and quantification [43]. Each polymer type produces a unique and reproducible pattern of pyrolysis products, allowing for wide range of identification [44,45].

The primary advantage of Py-GC-MS is its exceptional chemical specificity and its ability to provide quantitative, mass-based data [46,47,48]. Unlike spectroscopic methods that analyze particles one by one, Py-GC-MS can analyze an entire filter, providing a total mass of each polymer type present in the sample. This is invaluable for exposure assessment, as it moves beyond particle counts to actual mass concentration. The main limitations are that the technique is destructive, meaning all information about particle size, shape, and morphology is lost [42]. Furthermore, traditional Py-GC-MS systems have very low throughput for particle-based analysis, as samples often have to be introduced manually one at a time, making it unsuitable for routine monitoring of large numbers of individual particles [49,50].

### 5.3. Microscopic and Elemental Analysis: SEM-EDS

Scanning Electron Microscopy (SEM) is a critical technique for detailed characterization of the physical and surface properties of MPs particles. SEM uses a focused beam of electrons to scan the surface of a sample, generating high-resolution images with magnifications up to 500,000× [51,52]. This allows for detailed examination of particle morphology (shape), surface texture (e.g., signs of weathering or degradation), and precise size measurements, even at the nanoscale [53]. This level of detail is crucial for understanding the physical state of MPs recovered from biological tissues and can provide clues about their origin and history.

When coupled with an Energy-Dispersive X-ray Spectroscopy (EDS) detector, the SEM becomes a tool for elemental analysis. The electron beam excites atoms in the sample, causing them to emit characteristic X-rays whose energies correspond to specific elements [54]. SEM-EDS can thus map the elemental composition of a particle. While it cannot identify the organic polymer backbone (composed mainly of carbon and hydrogen), it is highly effective for identifying inorganic additives, fillers, or pigments within the plastic matrices [52,55]. It can also be used to confirm the identity of certain polymers that contain unique heteroatoms, such as the chlorine in PVC or the nitrogen in PA. However, because it does not provide molecular structure information, SEM-EDS is primarily considered a complementary technique to be used alongside vibrational spectroscopy or thermal analysis.

### 5.4. Emerging and Portable Detection Methods

While the aforementioned techniques form the current gold standard, they are typically lab-based, expensive, and require significant expertise. In response, research is moving toward the development of more accessible, rapid, and potentially portable detection methods. One prominent approach involves the use of fluorescent dyes, such as Nile Red, which selectively adsorb onto hydrophobic plastic surfaces [56,57,58]. When illuminated with the appropriate wavelength of light, the stained particles fluoresce, allowing for rapid counting and visualization via fluorescence microscopy. While this method is excellent for high-throughput screening, it can suffer from non-specific staining of natural organic matter and does not provide polymer identification [59].

Further along the innovation pipeline are sensor-based technologies designed for real-time or on-site analysis. These include electrochemical biosensors that can detect changes in electrical properties (e.g., impedance) as particles pass over an electrode, and plasmonic sensors that use nanostructured metal surfaces to enhance spectroscopic signals, such as in Surface-Enhanced Raman Spectroscopy (SERS) [60]. While these technologies show great promise for creating low-cost, portable devices, their application to complex biological matrices is still in the very early stages of research and development [61,62].

To facilitate informed selection of analytical methods for MPs characterization, Table 3 provides a structured comparison of the principal techniques currently employed in the field. The table outlines each method’s operating principle, the nature of information it yields such as polymer identity, particle morphology, or mass and critically evaluates their respective strengths and limitations, particularly in the context of complex biological matrices. This comparative overview serves as a practical reference for researchers aiming to align analytical capabilities with specific sample requirements and study objectives.

**Figure 4:**
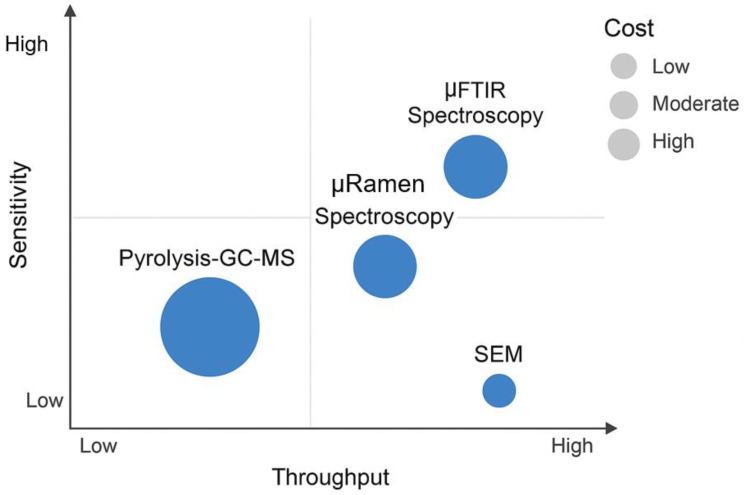
Comparative analysis of commonly used microplastic detection techniques based on sensitivity, throughput, and cost.

These tools vary significantly in their throughput, sensitivity, particle size resolution, and cost, which contributes to the current trade-offs in method selection and standardization (Figure 4).

## 6. Limitations, Trade-Offs, and Critical Gaps

Despite the availability of sophisticated analytical instrumentation, the field of MPs in biological matrices is restricted by a series of fundamental limitations and gaps. These challenges create significant trade-offs in experimental design and, most critically, contribute to a pervasive lack of standardization that impedes scientific progress. Addressing these gaps is essential for moving from qualitative detection to robust, quantitative risk assessment.

### 6.1. Balancing Sensitivity, Throughput and Cost in Microplastic Analysis

The current landscape of MPs analysis is constrained by a fundamental trade-off among three key performance parameters, sensitivity, analytical throughput, and cost, making it challenging to optimize all simultaneously within a single methodological framework. It is currently not possible to optimize all three simultaneously; an improvement in one typically comes at the expense of another. High-sensitivity techniques capable of detecting and identifying very small particles, such as µ-Raman spectroscopy, are characterized by low throughput (analyzing small areas or individual particles is time-consuming) and high capital and operational costs [69]. Conversely, methods that offer high throughput and low cost, such as visual microscopy or bulk fluorescent staining, lack the sensitivity and chemical specificity required for reliable identification, especially of small or colorless particles [70].

Thermal methods like Py-GC-MS offer high sensitivity for quantifying polymer mass but have extremely low throughput when considered on a per-particle basis and are also costly [71,72]. This forces researchers into difficult compromises. A study aiming for high statistical power by analyzing many samples may be forced to use a lower-cost, lower-sensitivity method, risking inaccuracy. A study prioritizing the detection of the smallest particles may be limited to analyzing only a few samples due to the time and expense involved, limiting the generalizability of its findings. This fundamental trade-off is a major constraint that hinders the large-scale data generation needed for comprehensive exposure and risk assessment.

### 6.2. The Lack of Standardization: A Barrier to Comparative Science

The most significant barrier to progress in the field is the lack of standardization across all stages of the analytical workflow [71,72]. Different research groups employ a wide variety of methods for sample collection, storage, pre-treatment, and analysis, making it exceptionally difficult, if not impossible, to compare results across studies. For example, one study might use KOH digestion and report particle counts per gram of wet tissue, while another uses enzymatic digestion and reports the mass of polymers per gram of dry tissue. These differences in sample processing can lead to varying polymer recovery rates, and the disparate reporting units prevent direct comparison [6].

This issue extends beyond procedural inconvenience and represents a critical barrier to scientific progress. In the absence of standardized protocols, it becomes difficult to perform reliable meta-analyses, establish consistent global baselines for human exposure, or generate robust, cross-validated datasets essential for epidemiological investigations [6]. This issue is further compounded by economic and infrastructural disparities. The “gold standard” instruments are expensive and require specialized expertise, concentrating high-quality research capabilities in well-funded institutions and regions. This creates a global inequity, where labs with fewer resources may be forced to rely on less reliable, non-standardized methods, perpetuating the cycle of data incomparability. Therefore, the call for standardization must be accompanied by the development of analytical technologies that are not only accurate but also accessible and affordable on a global scale.

### 6.3. The Nanoplastic Detection Gap

A critical and largely unaddressed gap is the detection and quantification of NPs in biological samples [73]. As particle size decreases into the sub-micrometer range, the analytical challenges escalate dramatically. Most widely used techniques, like FTIR, are fundamentally limited by the laws of physics (the diffraction limit of light) and cannot resolve individual nanoparticles [37,74]. While advanced microscopy (SEM, AFM) and spectroscopy (Raman, SERS) can visualize nanoscale objects, their application to complex biological digests presents significant technical challenges [75,76]. The signal from a single nanoparticle is extremely small and can be easily lost in the noise from the instrument or interference from the sample matrix.

Furthermore, there is a critical lack of environmentally relevant reference materials for NPs [77]. Method development and validation require well-characterized standards, but the spherical, pristine polystyrene nanoparticles often used in lab studies bear little resemblance to the irregularly shaped, weathered, and biofilm-coated NPs formed in the real world. This gap in our analytical capability is deeply concerning because these are the very particles theorized to have the highest bioavailability and potential for cellular and subcellular toxicity [78]. Until this NPs detection gap is closed, our understanding of human exposure and risk will remain fundamentally incomplete.

### 6.4. Contamination Control and Environmental Relevance

Two important practical issues that reduce the reliability of many studies are poor contamination control and disconnect from real-world environmental conditions. Because plastics are found everywhere in laboratory environments, there is a high risk that MPs particles may accidentally enter samples during handling and analysis [79,80]. Fibers from clothing, fragments from plastic labware, and particles from airborne dust can all be introduced during sample processing. Failing to implement and rigorously monitor strict quality control measures, such as the consistent use of procedural blanks, can lead to the reporting of false positives and a significant overestimation of MPs concentrations [81,82].

Additionally, there is often a significant disparity between the types of particles used in laboratory toxicology studies and those to which humans are actually exposed. Many experiments use commercially available, pristine, spherical polymer beads because they are uniform and easy to work with. However, the vast majority of MPs in the environment are secondary fragments that are irregularly shaped, have weathered and oxidized surfaces, and are often coated with a biofilm of microorganisms and adsorbed pollutants [15]. These properties can drastically alter their transport, bioavailability, and toxicity compared to pristine spheres. This gap in environmental relevance limits the direct applicability of many laboratory findings to real-world human health risk assessment.

## 7. Innovations and Future Directions

Overcoming the formidable challenges in MPs analysis requires a paradigm shift away from incremental improvements of existing methods toward the design of novel, integrated analytical systems. Addressing these challenges requires a multidisciplinary approach that integrates materials innovation, process miniaturization, and advanced data analytics. The future of MPs research will be shaped by the development of novel materials, the adoption of microscale analytical platforms, and the application of artificial intelligence to manage and interpret increasingly complex datasets with greater speed and precision.

### 7.1. Advanced Nanofiltration Based Membrane Technologies

While conventional microfiltration remains widely used for concentrating MPs, the detection and recovery of NPs require the implementation of more advanced membrane technologies. Nanofiltration (NF) membranes, with precisely engineered pore sizes in the nanometer range, offer a promising method for concentrating NPs from complex liquid samples, such as the final digestate of a biological tissue [83]. An effective workflow may involve a multi-stage membrane system, where microfiltration initially removes larger particles and debris, followed by ultrafiltration and NF steps to progressively concentrate smaller-sized particles, including NPs.

The primary challenge in implementing such a system is severe membrane fouling. Residual biomolecules such as peptides, lipids, and saccharides left over from an incomplete digestion process can rapidly adsorb to the membrane surface, blocking the pores and drastically reducing flux. Therefore, the successful application of NF is linked to advances in both upstream digestion efficiency and the design of novel, low-fouling membrane materials. Researchers are actively developing membranes with modified surface chemistries, such as hydrophilic or zwitterionic coatings, that resist the adsorption of biomolecules, a critical step toward making nanofiltration a viable tool for NPs concentration in biological samples.

### 7.2. Smart Materials: Selective Binding with Molecularly Imprinted Polymers and Functionalized Adsorbents

An advanced strategy for sample clean-up would shift from non-specific bulk removal of biological material to the selective isolation of target MPs particles. This can be achieved through the design of “smart” adsorbent materials with tailored surface chemistry. Among the most promising of these are Molecularly Imprinted Polymers (MIPs) [84]. MIPs are synthesized using a template-based approach where functional monomers are polymerized around a target molecule or structure. After polymerization, the template is removed, leaving behind recognition cavities that are sterically and chemically complementary to the template, creating a “molecular memory” [85].

**Figure 5:**
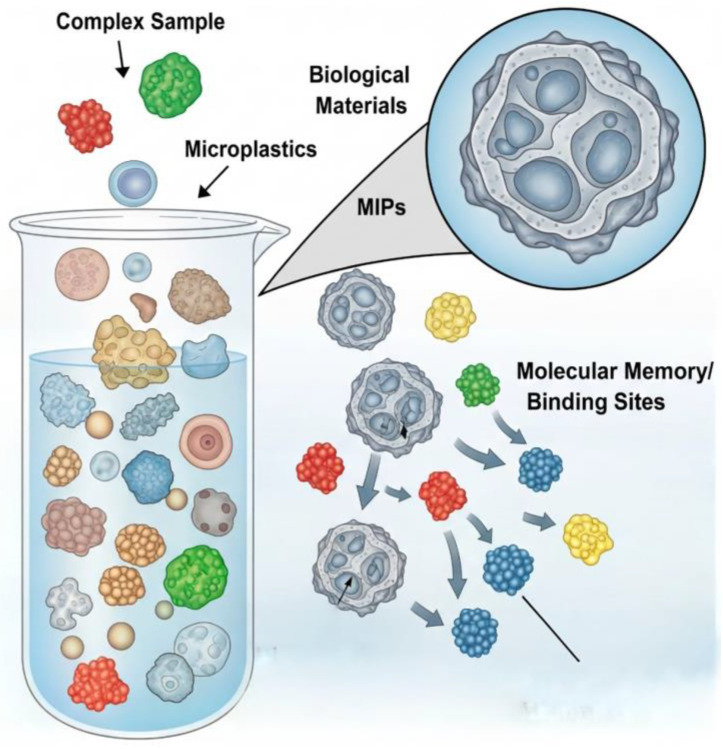
Selective isolation of microplastics using Molecularly Imprinted Polymers (MIPs).

While traditionally used for small molecules, the principles of surface imprinting can be extended to create MIPs that selectively recognize and bind to the surfaces of specific polymer particles, such as PS or PET [86]. These MIPs could be used as a solid-phase extraction medium to selectively pull MPs particles out of a complex biological digestate, leaving interfering biomolecules behind. This would dramatically improve sample purity and reduce matrix effects in subsequent analyses [84]. Other engineered adsorbents, such as magnetic nanoparticles functionalized with hydrophobic ligands or specific plastic-binding peptides, also offer pathways for selective, magnetically recoverable separation of MPs from aqueous solutions [87]. Figure 5 presents the visualization of the principle of surface-imprinted MIPs, which retain “molecular memory” through recognition cavities that selectively bind MPs particles. By distinguishing target polymers within a complex biological matrix, MIPs enable highly selective extraction and reduce matrix interference, improving downstream analytical accuracy.

### 7.3. Process Intensification: The Role of Microfluidic Separation and Analysis Platforms

The current bench-scale workflow for MPs analysis is labor-intensive, time-consuming, requires large volumes of reagents, and is highly susceptible to contamination. Process intensification offers a solution through the development of microfluidic “lab-on-a-chip” systems [88]. These devices use micro-fabricated channels to manipulate fluids and particles at the micrometer scale. By applying principles of laminar flow, inertia, and dielectrophoresis, microfluidic platforms can be designed to continuously sort particles by size and shape with high precision [89].

**Figure 6:**
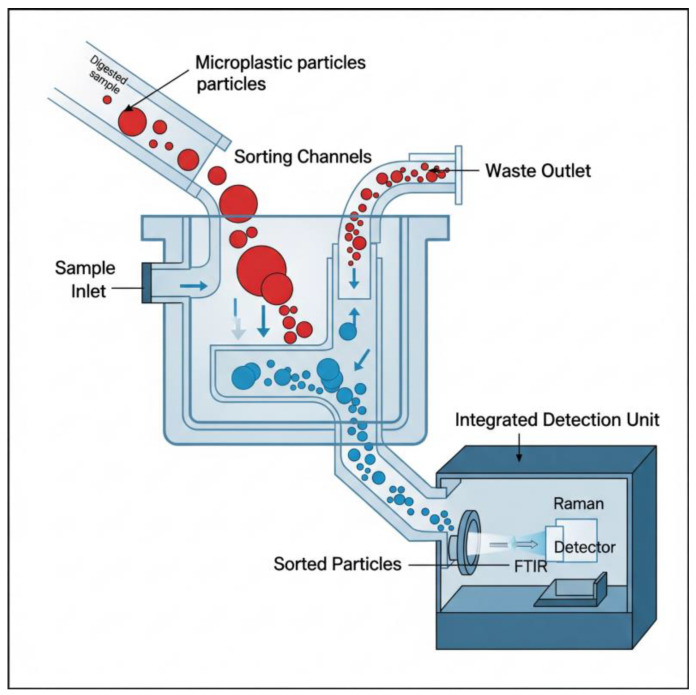
Microfluidic lab-on-a-chip system for sorting and detecting microplastic particles.

The key advantage of microfluidic technology lies in its ability to integrate multiple analytical processes within a single miniaturized platform. A single chip could be designed to perform an entire analytical workflow: receiving a digested sample, separating particles from the liquid, staining them with a fluorescent dye, and passing them through an on-chip detector for counting and sizing [88]. Such an integrated system would drastically reduce sample and reagent volumes, shorten analysis times from days to minutes, and minimize manual handling, thereby reducing the risk of contamination. This represents a shift from discrete, manual benchtop steps to a continuous, automated analytical process. The schematic (Figure 6) illustrates a microfluidic device where digested biological samples enter through a sample inlet. Sorting channels direct MPs particles based on size; smaller particles continue toward an integrated detection unit equipped with FTIR and Raman spectrometers, while larger particles exit through a waste outlet. This compact, automated system exemplifies process intensification and real-time analysis.

### 7.4. AI-Aided Spectral Classification and High-Throughput Analysis

The final step of the analytical workflow interpreting vast amounts of complex spectral data is a major bottleneck. Manually inspecting thousands of spectra from an FTIR or Raman map is prohibitively time-consuming and subjective. This is where Artificial Intelligence (AI) and Machine Learning (ML) are revolutionizing the field [90]. Researchers are now training sophisticated ML algorithms, particularly deep learning models like Convolutional Neural Networks (CNNs), on large libraries of polymer spectra [91]. These models can learn to recognize the subtle “fingerprint” patterns unique to each polymer type with extraordinary accuracy [92].

However, the feasibility of AI-assisted workflows in routine microplastic analysis remains constrained by several limitations. First, the accuracy of models is highly dependent on the representativeness of training datasets, which are often biased toward pristine reference spectra rather than environmentally weathered or biologically embedded plastics. Second, reproducibility across laboratories is limited, as differences in instrumentation, sample preparation, and preprocessing pipelines can produce spectral variations that confound AI models. Third, while AI excels at pattern recognition, the “black-box” nature of many models raises challenges for regulatory acceptance, where transparent and interpretable decision-making is essential. Finally, the integration of AI into standardized workflows requires significant computational resources and technical expertise, which may not be available in lower-resource laboratories.

**Figure 7:**
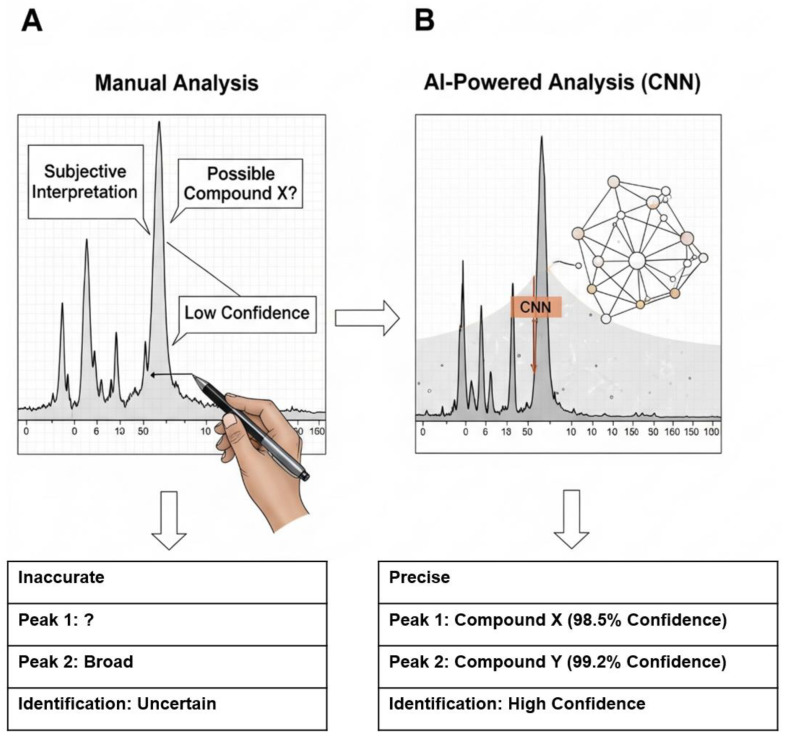
Comparison between traditional manual spectral identification and AI-automated classification using convolutional neural networks (CNNs).

By integrating these AI models into the analytical software, the process of spectral identification can be fully automated. The software can automatically process a spectral map containing thousands of data points, identify every polymer particle, classify its type, and measure its dimensions in a fraction of the time required for manual analysis [93]. This AI-driven approach not only provides a massive increase in throughput but also enhances objectivity and reproducibility by eliminating human error and bias [90]. Furthermore, machine learning algorithms can be trained to resolve complex spectra obtained from weathered, contaminated, or overlapping particles, enabling the extraction of meaningful information from non-ideal samples that would be challenging or impossible to interpret manually. Comparison between traditional manual spectral identification and AI-automated classification using convolutional neural networks (CNNs) is represented in Figure 7 [94].

### 7.5. Development of Integrated and Scalable Workflows for Advanced Microplastic Detection

The future of MPs analysis lies in adopting a holistic, systems-oriented approach that emphasizes the integration of all analytical stages rather than optimizing individual components in isolation. This shift is essential for building robust, reproducible, and scalable workflows capable of handling complex biological matrices [95]. The development of next-generation analytical platforms for MPs detection will require the integration of multiple scientific and technical principles. In particular, optimizing digestion protocols will demand a careful balance between effective removal of biological material and the preservation of polymer integrity, necessitating selective and efficient reaction conditions [96]. Advancements in materials research will be essential for developing selective membranes and engineered adsorbents capable of efficiently separating and concentrating MPs particles from complex matrices. Additionally, principles of fluid dynamics and particle transport will be instrumental in designing microfluidic systems that enable precise sorting and handling of particles based on their size and properties [95]. Finally, automation and data science play a vital role in integrating all components of the analytical workflow into cohesive, high-throughput systems. The application of artificial intelligence enables the rapid and accurate interpretation of complex spectral and imaging data, significantly enhancing the reliability and scalability of MPs detection [83,97].

**Figure 8:**
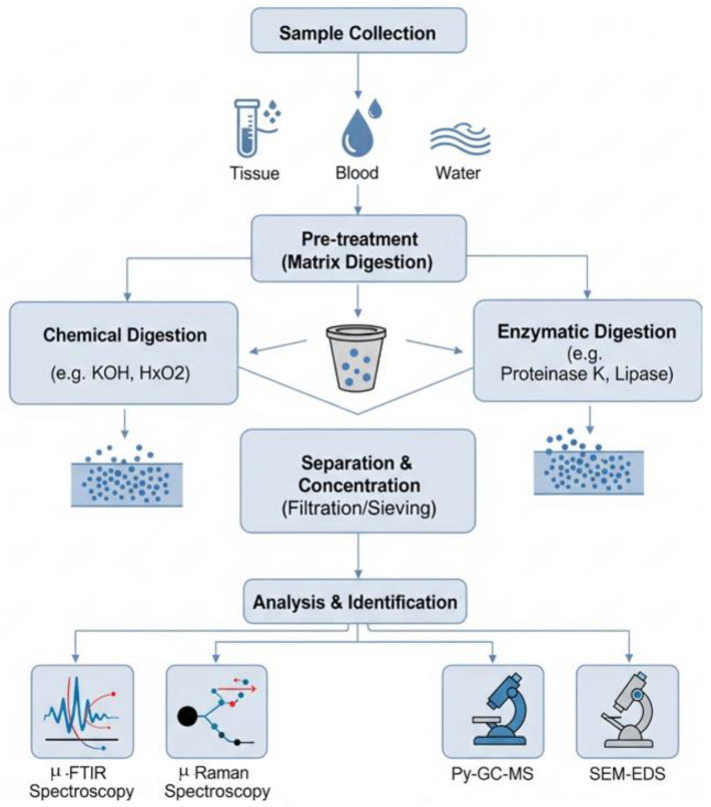
Schematic overview of the sample-to-answer workflow for microplastic detection in biological samples.

By viewing the entire sample-to-answer process as a unified analytical workflow, researchers can systematically identify bottlenecks, improve overall efficiency, and develop robust, scalable, and standardized methods that are critical for accurately assessing the health risks posed by MPs exposure. As illustrated in Figure 8, this workflow typically begins with the collection of biological samples such as blood, tissue, or stool. The next phase involves pre-treatment or digestion to remove interfering organic matter. Chemical digestion agents like KOH or H_2_O_2_ are commonly used due to their effectiveness, though they pose a risk of degrading certain sensitive polymers. In contrast, enzymatic digestion using agents such as proteinase K or lipase provides a gentler approach that better preserves polymer integrity, albeit with the potential drawback of incomplete removal of biomolecules.

Following digestion, separation and concentration of MPs are usually carried out through physical techniques such as filtration, sieving, or centrifugation. These methods help isolate particles from the digested matrix but differ in efficiency and particle size cut-off. The final stage of the workflow is analytical identification and quantification, employing high-resolution spectroscopic and imaging techniques. These include μ-FTIR, Raman spectroscopy, pyrolysis-GC-MS, and SEM-EDS, each offering unique advantages and limitations in terms of sensitivity, specificity, particle size detection, and throughput. The variability among these methods contributes to the current challenges in method harmonization, ultimately impacting the comparability and reliability of exposure assessments across studies.

Table 4 presents a summary of emerging analytical approaches designed to address key limitations in current MPs detection methods. It highlights the core scientific principles behind each innovation and evaluates their potential to enhance accuracy, sensitivity, and throughput in future MPs analysis workflows.

## 8. Conclusion and Outlook

### 8.1. Synthesis of the Current State-of-the-Art

MPs research has clearly demonstrated the widespread contamination of the environment and the consequent, unavoidable human exposure. Biomonitoring studies have confirmed the presence of various synthetic polymer particles in human tissues and fluids, while extensive toxicological evidence from experimental models supports concerns about their potential adverse health effects. However, as outlined in this review, translating these findings into a robust and quantitative human health risk assessment remains severely hindered by a set of interrelated analytical challenges. The current state of the field is constrained by a fundamental bottleneck, the lack of reliable, accurate, and efficient methods for isolating and detecting MPs, particularly NPs, from complex biological matrices.

Despite remarkable advancements in MPs research, current analytical workflows remain constrained by a complex interplay of methodological trade-offs. At the pre-treatment stage, researchers face a critical decision between aggressive matrix digestion and preservation of particle integrity. Harsh chemical or oxidative protocols, such as strong acids, alkalis, or peroxide-based digestion, are effective at removing complex biological materials, especially in lipid-rich or protein-dense matrices such as liver tissue or breast milk. However, these treatments pose a significant risk of altering or degrading target MPs particles, particularly those composed of more chemically sensitive polymers This degradation may result in both underestimation of particle abundance and mischaracterization of polymer composition, thereby compromising the reliability of downstream data.

In contrast, gentler pre-treatment strategies such as enzymatic digestion prioritize the preservation of polymer structure and morphology. These approaches use selective enzymatic reactions to break down specific biomolecules (e.g., proteins, lipids, or carbohydrates) without affecting synthetic polymers. While they offer improved polymer recovery and maintain sample integrity, their limited efficiency in completely removing biological material often results in residual matrix interference during spectroscopic or microscopic analysis. Incomplete digestion introduces noise and background signals, complicating the accurate detection and chemical identification of MPs particles, particularly those that are small, translucent, or heavily weathered.

These trade-offs extend into the detection phase, where another layer of compromise emerges between sensitivity, specificity, throughput, and cost. High-resolution and chemically specific techniques, such as Raman microspectroscopy, µ-FTIR, and pyrolysis-GC-MS, offer excellent sensitivity and the ability to discriminate between polymers at the micrometer scale. However, these methods are time-intensive, laborious, and require significant technical expertise. Their low throughput and high operational cost render them impractical for large-scale epidemiological studies or routine environmental health monitoring. Additionally, the need for extensive sample preparation, precise calibration, and the risk of operator bias further complicate their adoption in standardized workflows. On the other end of the spectrum, high-throughput screening methods such as Nile Red fluorescence staining or bulk particle imaging provide faster results and are more accessible to lower-resource laboratories. However, these techniques often lack the chemical specificity required for definitive polymer identification and are prone to overestimating particle counts due to non-specific binding or the inclusion of natural organic matter. Thus, they serve as useful preliminary screening tools but are inadequate for quantitative exposure assessment or regulatory decision-making.

Adding to these technical challenges is the persistent and widespread lack of methodological standardization across laboratories and studies. There is currently no universally accepted protocol for sample collection, storage, digestion, separation, or analysis. Consequently, studies employ varying approaches, report data in inconsistent units and utilize different detection thresholds. These discrepancies severely hinder cross-comparability, obstruct the aggregation of findings into meaningful meta-analyses, and prevent the establishment of global baselines for human MPs exposure. Without harmonized procedures, the scientific community cannot build robust, reproducible datasets essential for informing public health policies or setting regulatory limits.

Addressing these challenges requires not only the development of more sensitive analytical tools but also an integrative approach that balances sample preparation, separation, detection, and data analysis within a coherent and standardized workflow. Without such a unified framework, the field will remain fragmented, and the potential health implications of micro- and NPs exposure will remain poorly understood and inadequately managed.

### 8.2. Advancing Microplastic Analysis Through Integrated Separation and Analytical Strategies

Addressing the current bottlenecks in MPs analysis requires more than incremental improvements to existing methods. It demands a fundamental rethinking of the analytical workflow as an integrated, end-to-end system. The challenge of isolating trace-level particles from complex biological matrices is, at its core, an advanced separation problem that must be approached with precision, scalability, and analytical rigor.

The way forward lies in the design of unified analytical platforms that are not only efficient and sensitive but also robust, automated, and reproducible across diverse sample types. Such systems must integrate innovations across multiple scientific domains: advances in materials science to develop highly selective adsorbents and anti-fouling membranes; optimized digestion strategies that balance matrix removal with polymer preservation; and microfluidic systems capable of sorting particles by size, shape, or surface chemistry with high resolution. Equally critical is the role of data science and automation in accelerating analysis and ensuring reproducibility. Emerging tools in AI and ML can be employed to automate spectral classification, reduce human bias, and improve the accuracy and throughput of MPs identification.

By treating the sample-to-answer pipeline as a coherent analytical system to be designed, integrated, and optimized, researchers can overcome the current limitations that fragment the field. This systems-level perspective is essential for enabling the development of next-generation detection workflows that are adaptable, high-throughput, and suitable for global monitoring efforts. Such progress will ultimately pave the way for more accurate exposure assessments and evidence-based policy interventions to mitigate MPs-related health risks.

While the present review has assessed pre-treatment, separation, and detection techniques individually, it is increasingly evident that the future of MPs/NPs biomonitoring lies in integrated analytical workflows. For example, sequential use of enzymatic digestion followed by density separation and spectroscopic confirmation can reduce false positives while enhancing recovery rates across different matrices. Similarly, coupling microfluidic pre-concentration with Raman or FTIR spectroscopy offers a scalable strategy to improve sensitivity, particularly for NPs. Such hybrid or sequential approaches have the potential to maximize accuracy and efficiency, and their broader adoption will be critical to translating current laboratory techniques into robust biomonitoring pipelines applicable across diverse human matrices.

### 8.3. A Call for Multidisciplinary Research and Standardization

Certain populations face disproportionate risks from MPs exposure. For example, workers in plastic manufacturing, recycling, and textile industries are routinely exposed to elevated concentrations of airborne MPs and fibers, which may lead to chronic respiratory and systemic effects. Waste handlers and incinerator workers in low-resource settings represent another vulnerable group with potentially high exposures but limited biomonitoring data. Ethical considerations therefore demand that future research prioritize these occupationally and socioeconomically vulnerable populations, both to protect worker health and to establish exposure limits relevant for regulatory guidelines.

From a technological standpoint, scalable detection in real-world settings will likely require hybrid analytical approaches that integrate the strengths of complementary techniques. For instance, coupling microfluidic separation platforms with Raman or FTIR spectroscopy can enable automated, high-throughput particle isolation and polymer identification within complex biological matrices. Similarly, combining AI-based spectral classification with microfluidics offers the potential to minimize operator subjectivity while dramatically improving throughput. Such hybrid approaches are essential to move beyond proof-of-concept demonstrations toward robust, standardized pipelines that can be implemented in biomonitoring programs at both clinical and environmental health levels.

The primary objective of MPs research is to safeguard human health. Achieving this goal requires a coordinated, multidisciplinary effort that bridges the gaps between analytical science, toxicology, environmental health, and public policy. While advanced tools and technologies are critical for improving detection and characterization, their development must be closely aligned with the informational needs of toxicologists, environmental scientists, and public health professionals. Active collaboration across these domains is essential to ensure that analytical methods are not only scientifically rigorous but also capable of producing biologically meaningful and clinically relevant data. This includes improving techniques to assess particle weathering, detect adsorbed contaminants, and investigate interactions at the particle–biomolecule interface, as these factors are critical for understanding toxicity, biological transport, and long-term health implications.

Beyond analytical advances, the issue of microplastic detection in humans also carries significant ethical and policy implications. The lack of standardized biomonitoring protocols currently hampers comparability between studies, obscuring true global exposure trends. Without harmonized methodologies, populations in regions with the greatest exposure risk—often low- and middle-income countries with limited waste management infrastructure—remain underrepresented in the evidence base. From an ethical standpoint, this introduces inequities in both recognition and mitigation of health risks. Standardized, validated protocols for human biomonitoring are therefore essential not only for scientific reproducibility but also for informing equitable public health guidelines. International coordination led by organizations such as the World Health Organization (WHO) and the United Nations Environment Program (UNEP) will be critical to establish minimum standards, ensure global data comparability, and support the development of health-protective regulations that are responsive to diverse socio-economic contexts.

**Figure 9:**
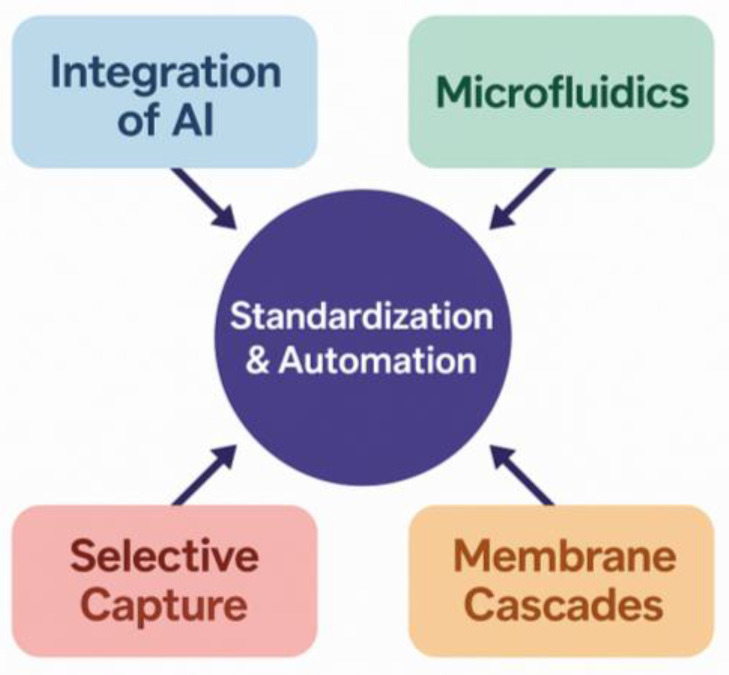
Key technological innovations envisioned to advance microplastic analysis.

Finally, this review reinforces the widespread consensus within the scientific community on the urgent need to develop and globally implement standardized and validated protocols for MPs analysis in human samples. As shows in Figure 9, the establishment of standard reference materials, harmonized sample preparation procedures, and uniform data reporting formats is an absolute prerequisite for building the high-quality, comparable datasets needed to conduct robust epidemiological studies. Only through such a standardized, multidisciplinary, and engineering-driven approach can the scientific community hope to definitively bridge the gap between measuring MPs contamination and truly understanding its impact on human health, thereby providing the evidence base needed for effective policy and mitigation strategies.

## Figures and Tables

**Table 1:** Characteristics of Microplastics in Human Exposure Pathways.

Exposure Pathway	Common Polymer Types	Typical Particle Sizes and Morphologies	Primary Sources
Ingestion	Polyethylene (PE), Polypropylene (PP), Polystyrene (PS), Polyethylene terephthalate (PET), Polyvinyl chloride (PVC) [12].	Wide range (10 µm–5 mm) [21]. Fragments and spheres are common. Particles ≤10–20 µm are most likely to translocate [22].	Contaminated drinking/bottled water, seafood, salt, sugar, food packaging, take-out containers, baby bottles, agricultural soil contamination [3].
Inhalation	Polyester (PET), Nylon (PA), Acrylic, Polyvinyl chloride (PVC), Styrene–butadiene rubber (from tires) [21].	Primarily fibers and small fragments. Aerodynamic diameter determines deposition; smaller particles (<10 µm) reach deep lung tissue [17].	Synthetic textiles, clothing, carpets, tire and road wear particles, industrial emissions, waste incineration [13].
Dermal/Systemic	Varied, including PE, PVC, PET from medical devices.	Varied. Particles from 1 µm to 62 µm detected in IV fluids [19].	Cosmetics, synthetic clothing (dermal). Intravenous (IV) bags, catheters, and other medical devices (systemic) [19].

**Table 2:** Comparative Analysis of Pre-treatment Methods for Biological Matrices.

Method	Principle of Action	Typical Digestion Efficiency	Polymer Compatibility/Integrity	Pros	Cons
Alkaline Digestion (e.g., 10% KOH)	Saponification of lipids and hydrolysis of proteins.	High (>90%) for many tissues [40].	Poor. Known to degrade or damage sensitive polymers like PET, PC, and cellulose acetate [24].	Fast, low-cost, highly effective for fatty tissues [40].	Destructive to certain key polymers, leading to biased results [24].
Oxidative Digestion (e.g., 30% H_2_O_2_, Fenton)	Radical oxidation of organic matter.	High (93–96%) [35].	Moderate. Generally safer than strong acids/bases, but can damage polymers like PA at elevated temperatures [24].	Effective on a wide range of organic materials; relatively inexpensive [35].	Can be aggressive; requires careful temperature control; safety concerns with concentrated reagents [24].
Acid Digestion (e.g., HNO_3_)	Strong acid hydrolysis and oxidation of all organic matter.	Very High (>95%) [24].	Poor. Highly destructive to many polymers, particularly PA [24].	Extremely effective at removing organic matrix.	Highly corrosive and hazardous; significant risk of polymer degradation [24].
Enzymatic Digestion (e.g., Proteinase K, Trypsin, Lipase)	Specific catalytic hydrolysis of target macromolecules (proteins, lipids).	Moderate to High (60–97%) depending on enzyme cocktail and matrix [35].	Excellent. Generally non-destructive and preserves the integrity of most synthetic polymers [35].	Highly specific and gentle on polymers; minimizes analytical artifacts [41].	Slow (hours to days); expensive; may require multiple enzymes for complex matrices; digestion may be incomplete [25].

**Table 3:** Comparison of Mainstream Analytical Techniques for Microplastic Identification.

Technique	Principle	Information Provided	Lower Size Limit	Strengths	Weaknesses
µ-FTIR Spectroscopy	Infrared light absorption by molecular bonds.	Polymer type, size, shape, chemical imaging.	~10–20 µm (Diffraction-limited) [63].	Robust, fast mapping with FPA detectors, extensive spectral libraries, non-destructive [64].	Poor spatial resolution for small MPs/NPs; water interference; sample must be dry [65].
µ-Raman Spectroscopy	Inelastic scattering of laser light by molecular bonds.	Polymer type, size, shape, chemical imaging.	~1 µm (can be <500 nm with advanced methods) [29].	High spatial resolution for small MPs/NPs; non-destructive; minimal water interference [66].	Fluorescence interference from biological residues/dyes; can be slower for large area mapping; potential for sample heating [27].
Py-GC-MS	Thermal decomposition followed by chromatographic separation and mass analysis.	Polymer type (mass concentration), additives.	No particle size limit (mass-based).	High chemical specificity; excellent for quantification of polymer mass; can analyze complex mixtures [67].	Destructive (loses all morphological data); low throughput for particle analysis; potential for overlapping pyrolysis products [29].
SEM-EDS	Electron beam imaging and characteristic X-ray emission.	High-resolution morphology, surface texture, size, elemental composition.	Nanometer-scale imaging.	Unparalleled imaging resolution; provides detailed physical characterization and degradation state; identifies inorganic additives [68].	Does not identify polymer structure; requires conductive sample (may need coating); quantitative analysis is complex [54].

**Table 4:** Emerging Technologies for Microplastic Separation and Detection.

Emerging Technology	Core Principle	Key Limitation Addressed	Potential Impact
AI-Aided Spectroscopy	Automated Process Control/Data Science	Low throughput, human error, and subjectivity in spectral analysis.	Fully automated, objective, and high-throughput polymer identification with high accuracy; analysis of weathered/complex particles [90].
Microfluidic Platforms	Process Intensification/Transport Phenomena	Large sample/reagent volumes, low throughput, high risk of contamination from manual handling.	Miniaturized, integrated “sample-to-answer” systems that automate separation, staining, and detection, increasing speed and reducing costs [88].
Smart Adsorbents (e.g., MIPs)	Materials Science/Surface Chemistry	Poor selectivity of separation; persistent interference from complex biological matrices.	Highly selective pre-concentration of target polymers, enabling cleaner samples for analysis and reducing matrix effects [84].
Advanced Mass Spectrometry (e.g., SRS, ToF-SIMS)	Advanced Analytical Instrumentation/Optics	Inability to detect and chemically identify individual nanoplastics in complex samples.	Routine chemical imaging and identification of particles < 1 µm, closing the critical nanoplastic detection gap [83].

## Data Availability

No new data were created or analyzed in this study.
